# Cutaneous Leishmaniasis: The Complexity of Host's Effective Immune Response against a Polymorphic Parasitic Disease

**DOI:** 10.1155/2019/2603730

**Published:** 2019-12-01

**Authors:** Áurea Gabriel, Ana Valério-Bolas, Joana Palma-Marques, Patrícia Mourata-Gonçalves, Pedro Ruas, Tatiana Dias-Guerreiro, Gabriela Santos-Gomes

**Affiliations:** Global Health and Tropical Medicine (GHTM), Instituto de Higiene e Medicina Tropical (IHMT), Universidade Nova de Lisboa (UNL), Rua da Junqueira 100, 1349-008 Lisboa, Portugal

## Abstract

This review is aimed at providing a comprehensive outline of the immune response displayed against cutaneous leishmaniasis (CL), the more common zoonotic infection caused by protozoan parasites of the genus *Leishmania.* Although of polymorphic clinical presentation, classically CL is characterized by leishmaniotic lesions on the face and extremities of the patients, which can be ulcerative, and even after healing can lead to permanent injuries and disfigurement, affecting significantly their psychological, social, and economic well-being. According a report released by the World Health Organization, the disability-adjusted life years (DALYs) lost due to leishmaniasis are close to 2.4 million, annually there are 1.0–1.5 million new cases of CL, and a numerous population is at risk in the endemic areas. Despite its increasing worldwide incidence, it is one of the so-called neglected tropical diseases. Furthermore, this review provides an overview of the existing knowledge of the host innate and acquired immune response to cutaneous species of *Leishmania.* The use of animal models and of *in vitro* studies has improved the understanding of parasite-host interplay and the complexity of immune mechanisms involved. The importance of diagnosis accuracy associated with effective patient management in CL reduction is highlighted. However, the multiple factors involved in CL epizoology associated with the unavailability of vaccines or drugs to prevent infection make difficult to formulate an effective strategy for CL control.

## 1. Introduction

Leishmaniases are anthroponotic and zoonotic diseases of global public health significance caused by obligatory intracellular digenetic parasites of the genus *Leishmania* [1–3]. These parasites are transmitted to human beings and mammalian hosts by the bite of infected sand flies of the *Phlebotomus* genus in the Old World and *Lutzomyia* in the New World, generating cutaneous or visceral leishmaniasis [4, 5]. More than 20 *Leishmania* species have been identified worldwide, according to the WHO [1, 5]. Several species of *Leishmania*, belonging to both *Leishmania* and *Viannia* subgenus, cause CL in humans, including *L. tropica*, *L. major*, and *L. aethiopica* in the Old World and also *L. mexicana*, *L. amazonensis*, *L. venezuelensis*, *L. braziliensis*, *L. shawi*, *L. guyanensis*, *L. panamensis*, and *L. peruviana* that are only found in the New World [2, 5, 6] (Table 1). Differences among *Leishmania* species can lead to diverse clinical manifestations and therapeutic responses [7–9]. The knowledge about the complex interactions between these species and the respective hosts, their geographical distribution, histopathological effects, clinical lesions, and immune evasion still need to be deepened [2, 4, 5, 7]. In general, cutaneous species cause skin and mucous membrane lesions, which can persist for a long time in patients suffering from the disease and can also relapse during treatment [10–12]. Some CL patients can develop permanent injuries, which can leave them disfigured and stigmatized for life [11, 13, 14]. Thus, this review is aimed at providing a comprehensive outline of the immune response generated against the cutaneous species of *Leishmania*, evidencing the need for further studies able to deepen the understanding of protective immune mechanisms and pointing out opportunities that might be explored to further reduce CL threat.

## 2. Cutaneous Leishmaniasis Has a Wide Geographic Distribution and Present Polymorphic Clinical Features

Cutaneous leishmaniasis is considered the most common form of a *Leishmania* infection, affecting approximately 0.7 million to 1.2 million human beings [1, 14, 15]. This clinical form is prevalent in more than 90 countries with a proven endemic transmission in tropical and subtropical areas of the world, including rural, rainforests, arid areas, semiurban, and urban areas [4, 15, 16]. According to Maia-Elkhoury et al. [16], increased number of cases may be attributed to behavioral and environmental changes, determined mainly by climate, social, and economic conditions that influence *Leishmania* transmission.

**Figure 1:**
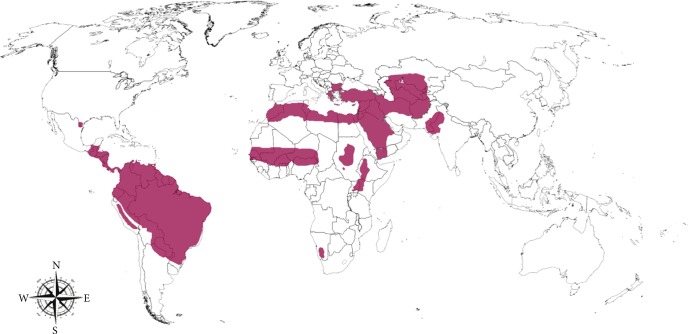
Worldwide distribution of cutaneous leishmaniasis, adapted from [1, 15, 18].

CL is present in the southern USA, where occasional cases were reported in the States of Texas and Oklahoma, Central and South America, being the majority of CL cases reported in Brazil and Peru [16–18], and in the Old World, at North and East Africa, Middle East, and Western and South Asia [18–20] (Figure 1). In these areas, some cities show very high notification rates for new CL cases, like Aleppo (Syria, Western Asia) with around 12.000 new cases each year [19–21].

*Leishmania* lesions without pain or pruritus are common, but in some patients can be painful, especially if ulcerative lesions become secondarily infected with bacteria or if these lesions are near a joint [22]. CL may range between a limited form, presenting only one or few localized lesions, to a disseminated form with multiple lesions (Table 2), including hypodermal, verrucous, sporotrichoid, impetigoid, hemorrhagic, erysipeloid, chancriform, lupoid, papular, psoriasiform, and ulcer-crusted lesions [11, 23, 24].

The lesions may start out as nodules in approximately 20% of cases during acute infections and persist in chronic infection [1]. Depending on the clinical type and stage, the epidermis may be overlying a dense dermal infiltrate, containing predominantly histiocytes, lymphocytes, and plasma cells [25]. Several patients with American CL may develop regional lymphadenopathy, occasionally bubonic, nodular lymphangitis (sporotrichoid-like subcutaneous nodules), and satellite lesions [1].

In epidemic regions of Western Asia, where cases of *L. tropica* advanced to aggressive and prolonged disease courses, the lesions impinged and possibly hindered the function of vital sensory organs, including olfactory perception and vision [19].

## 3. Competence of Innate and Acquired Immune Response Determines Infection Outcome and Cutaneous Leishmaniasis Severity

The dissemination and persistence of *Leishmania* parasites in the immunocompetent host depends on continuous parasite strategies able to modulate and subvert innate and adaptive immune response [25–27]. According to *in vitro* studies, the host genetic background, *Leishmania* species, and different parasite isolates can influence immune response [28]. Increasing interest in studying the immune response against cutaneous species of *Leishmania* in different animal models (such as susceptible BALB/c mice, resistant C57BL/6 mice, and nonhuman primates) has contributed to an improved understanding of specific parasite-host interactions and highly complex pathways of immune mechanisms underlying CL immunopathology [25, 29, 30]. However, a full understanding of the immune mechanisms that are activated or inactivated in CL patients is crucial for the reduction of disease incidence to a level that would have a minimal impact on public health.

Immune defense is characterized by two principal mechanisms, the innate immune response that is activated early during the primary stage of the infection and the adaptive immune response, which is the second line response. The bridge between these two responses is accomplished by antigen-presenting cells (APCs) and by cytokines released into the microenvironment by effector immune cells.

### 3.1. Inactivation of a Complement System Seems to Favor the Establishment of Parasite Infection

The complement system plays a critical role in the innate immune defense. Plasma proteins that constitute the complement system are implicated in the activation of classical (CP), alternative (AP), and lectin pathways (LP). Complement activation triggers the stimulation of proteolytic cascades, generating different molecules, such as anaphylatoxins, opsonins, and the membrane attack complex. At the end of the complement cascade, pathogens undergo lysis and opsonization and also an inflammatory response occurs.

*In vitro* studies have shown that noninfective *Leishmania* promastigotes are susceptible to complement-mediated lysis and that infective metacyclic promastigotes can actively resist [31].

Once in the dermis of mammals, infective promastigotes activate the complement system, and then, the parasite first survival mechanism comes into action, inhibiting complement cascade [32, 33].

Manipulation of the complement cascade is achieved through the inactivation of opsonins to promote macrophage (M*Φ*) attraction [26, 30]. Previous studies have shown that LP is efficiently activated, since mannose-binding lectin, a protein that binds to the lipophosphoglycan of several microorganisms, including *Leishmania*, initiates the proteolytic cascade causing pathogen lysis [33–35]. Moreover, LP activation triggers C3-convertase that converts C3 in C3b directing AP activation [34, 36].

Infective *Leishmania* promastigotes have developed mechanisms to subvert AP activation [37].

**Figure 2:**
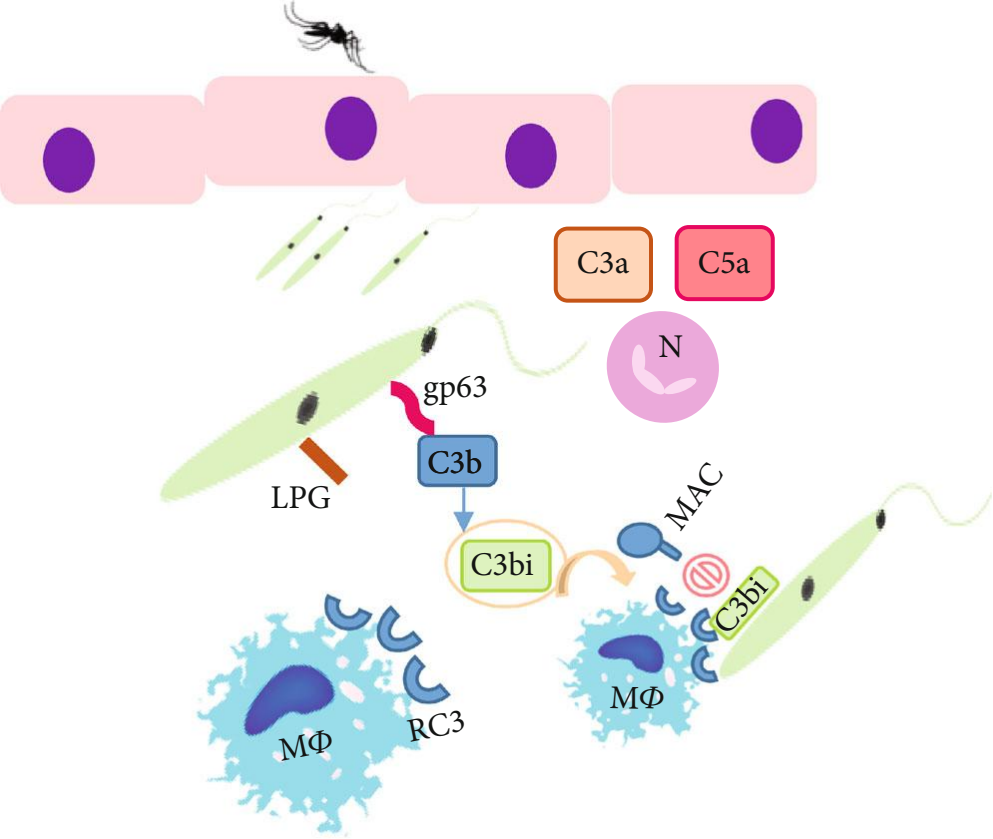
Inoculation of *Leishmania* promastigotes in the host dermis by the sand fly triggers the activation of the complement cascade. C3 convertase undergoes proteolytic cleavage, giving origin to the complement factors C3a and C5a. These chemotactic factors attract neutrophils (N) and macrophages (M*Φ*) to the infection site and induce the expression of the respective receptors. On the parasite membrane, the surface glycoprotein of 63 kDa (gp63) can convert C3b to the inactive form (C3bi), which avoids the assembly of the lytic complex (MAC) on the parasite surface. In turn, C3bi binds to the M*Φ* receptor (RC3), promoting fast parasite phagocytosis.

It was early found that the *Leishmania* surface glycoprotein captures C3b molecules [26, 30]. Upon the binding of C3 molecules to the glycoprotein of 63 kDa (gp63), C3b becomes inactivated (iC3b), which prevent the generation of C3-convertase [37]. iC3b that remains attached to the parasite surface is recognized by the complement receptor-3 that triggers promastigote phagocytosis by M*Φ* (Figure 2). Once uptake by M*Φ*, parasite differentiates into the amastigote form, which have the right conditions to initiate replication [26, 30].

Complement activation by *L. mexicana* membrane components was reported to cause the depletion of complement factors [37]. Complement exhaustion can drive the complement-independent parasite uptake by polymorphonuclear neutrophil granulocytes (PMNs), which can prolong parasite survival [30, 32]. Thus, the innate immune response drove by the complement system of the host seems to negatively impact on CL caused by *L. mexicana.*

Even so, previous studies in BALB/c mice showed that complement can diminish the spreading of *L. amazonensis* parasites in cutaneous lesions [38] and *in vitro* studies revealed that *L. tropica* amastigotes were susceptible to complement lysis [39], suggesting that during infection amastigotes can be susceptible to the complement system.

### 3.2. Neutrophils Seem to Have a Dual Effect Delaying the Early Establishment of Infection and Later One Favoring Lesion Pathology

Shortly after mammal infection by sand fly inoculation, infective metacyclic promastigotes have to evade host innate immunity to survive [25, 30].

Polymorphonuclear neutrophils (PMNs) are the first host cells that migrate to the site of *Leishmania* infection (as well as tissue M*Φ*), probably in response to sand fly saliva [40–42] that is inoculated together with parasites. These short-lived cells armed with a set of intracellular and extracellular mechanisms can arrest and kill pathogens [30, 32].

When encountering *Leishmania*, PMNs can internalize the parasite, generate an array of intracellular and extracellular microbicidal factors, such as reactive oxygen species, exocytosis of granule content rich in serine proteases that can damage the parasite membrane, and also emit web-like sticky structures (neutrophil extracellular traps (NETs)) to the extracellular space which can entrap and inactivate parasites, producing a proinflammatory environment [30–32]. However, despite the diverse mechanisms that PMNs have to contain pathogens, *Leishmania* parasites can survive, establishing infection and causing disease [30, 32, 33].

In the model of PMN intracellular infection, neutrophils can be used as “Trojan horses” assuring parasite survival and internalization by macrophages (M*Φ*), the definitive host cells, which also avoid the activation of M*Φ* killing mechanisms. Other studies performed with *L. major* revealed that mouse apoptotic PMNs can release viable parasites in the vicinity of surrounding M*Φ*, favoring parasite uptake by M*Φ* (Trojan rabbit mechanism) [28, 43].

The role of PMNs in controlling the dissemination of parasites at the early phase of cutaneous *Leishmania* infections has been studied *in vitro* and in experimental animal models [32]. An *in vitro* study showed that mouse neutrophils exposed to *L. guyanensis*, *L. shawi*, and *L. amazonensis* produced superoxide, released enzymes in the extracellular space, and generated NETs. However, *L. guyanensis* and *L. shawi* inhibited enzymatic activity and *L. amazonensis* reduced the NET emission, pointing towards the modulation of PMN extracellular effector mechanisms by cutaneous species of *Leishmania* [32]. Recent studies performed in the mouse model showed that PMN depletion accelerated the spreading of *L. major* and *L. amazonensis* parasites, leading to a more severe foot-pad swelling, which indicates that PMNs have a role in restraining parasite infection and in controlling the development of cutaneous lesions [44, 45]. Neutrophils seem to recognize these parasites through pattern recognition receptor- (PRR-) dependent mechanisms, such as toll-like receptor (TLR) 2, thereby activating downstream pathways that could compromise parasite survival [32, 46, 47].

After *in vitro* parasite stimulation, bloodstream PMNs from *L. braziliensis*-symptomatic patients were not more microbicidal than PMNs obtained from healthy subjects but presented a predominately proinflammatory profile, possibly influencing microenvironment and leukocyte recruitment [27, 47]. When in contact with parasites, PMNs isolated from healthy blood donors and from patients with American CL released NETs that contained and retained parasites, promoting its destruction as well as stimulated M*Φ* activity in order to control parasite infection [48, 49]. It was also verified *in vitro* that the interaction of *L. amazonensis*-exposed apoptotic or necrotic neutrophils with M*Φ* drove the initial M*Φ*-parasite infection, determining the infection outcome [50]. Taken together, these findings highlight the crucial role of PMNs in early infection, controlling cutaneous *Leishmania* parasites and delaying infection establishment and the progress of lesions [32, 50].

Neutrophils are present in unhealing cutaneous lesions, and recent studies performed in both humans and mice have shown that PMN infiltrates in cutaneous lesions induce immune-mediated tissue pathology [28, 40, 51].

Furthermore, PMN can present heterogeneous functional activity against different cutaneous *Leishmania* species and within the same species, indicating specific parasite immune recognition [9]. Different *L. braziliensis* isolates can promote specific activation of human PMNs. PMNs can inactivate *Leishmania* parasites or can favor infection, hosting viable infective parasites that can be delivered to the host cell. These findings support the hypothesis that PMNs can select the most infective parasites and inactivating the less virulent ones, which can generate novel avenues to explore the development of strategies underlying the modulation of PMN recruitment and activity that can direct lesion healing [52].

Although widely recognized as not having a key role in parasitic diseases caused by protozoa, *in vivo* studies highlighted eosinophil recruitment to *L. amazonensis* and *L. major* lesions [53–55]. It was also described that *L. braziliensis* patients that were in the early phase of lymphadenopathy exhibited cellular infiltrates enriched in eosinophils. [53]. Moreover, in *L. mexicana* early infection, eosinophils have been observed within the proximity of degranulating mast cells at the parasite inoculation site [56], pointing towards the occurrence of crosstalk between these two cells that could favor parasite clearance.

### 3.3. Cytotoxic Innate Cells Can Aid in the Control of Dermal Infection

After PMN recruitment, natural killer (NK) cells are also recruited in the early stage of *Leishmania* infection [25, 31]. NK cells are large granular leukocytes that play a key role in the innate immune response [57]. These cells are crucial in defining disease severity, restricting early parasite dissemination, and mediating direct lysis of parasitized cells, conferring protective immune response against *Leishmania* infection. Studies performed in the mouse model with *L. amazonensis* and *L. major* show the increase of parasite burden as a direct consequence of NK cell depletion [58–60]. These cells early release proinflammatory cytokines, such as interferon- (IFN-) *γ* that favors the differentiation of CD4^+^ Th1 cells and together with tumor necrosis factor- (TNF-) *α* activates the M*Φ* killing machinery [33, 59, 60]. However, it seems that *Leishmania* parasites have some mechanisms directed to suppress NK activity [58–60].

Studies performed in *L. major*-infected mice have demonstrated that NK cells exhibit a strong activation that peaks at 12 h to 48 h after infection, after which a steady decline tends to occur [61]. Some authors associate this suppression with the ability of this parasite to inhibit the production by neutrophils of NK cell-attracting chemokine IP-10 (CXCL9), which can prevent the activation of NK cells, therefore avoiding a continuous onset of NK cells from the bloodstream [62]. Furthermore, it was demonstrated that *L. major* promastigotes, the respective crude antigen, and gp63 can inhibit IFN-*γ* production by NK cells. gp63 seems to be able to bind to human NK cells, inhibiting cell's ability to produce IL-2 and downregulate some of the NK cell receptors, such as CD16 and CD56 [63]. In the case of *L. tropica* and *L. amazonensis* amastigotes, suppression of NK cell activity seems to be associated with low levels of IL-12. This interleukin released by DC determines NK cell activation. In human beings, it has been demonstrated an increase of CD56^+^NK cells in sites of healing lesions [64], thus suggesting a protective role of these cells in human leishmaniasis.

Although it was demonstrated that NK cells protect against CL, some studies indicate that these cells play a minor role [31, 53]. Thus, it is possible that NK cells can aid the immune system fighting against infection, but the involvement of these cells is still poorly studied in human CL and remains controversial in experimental murine leishmaniasis [53, 54, 65]. However, NK cell activity differs between patients with *L. mexicana* localized (LCL) and diffuse (DCL) CL. Reduced NK cell numbers in DCL patients associated with TLR downexpression and low cytokine production can be related to disease severity [65].

Therefore, it is possible that the regulation of NK cells can lead to a new opportunity for targeting CL control.

### 3.4. Antigen-Presenting Cells Have a Key Role in Directing T Cell Effector Activity

**Figure 3:**
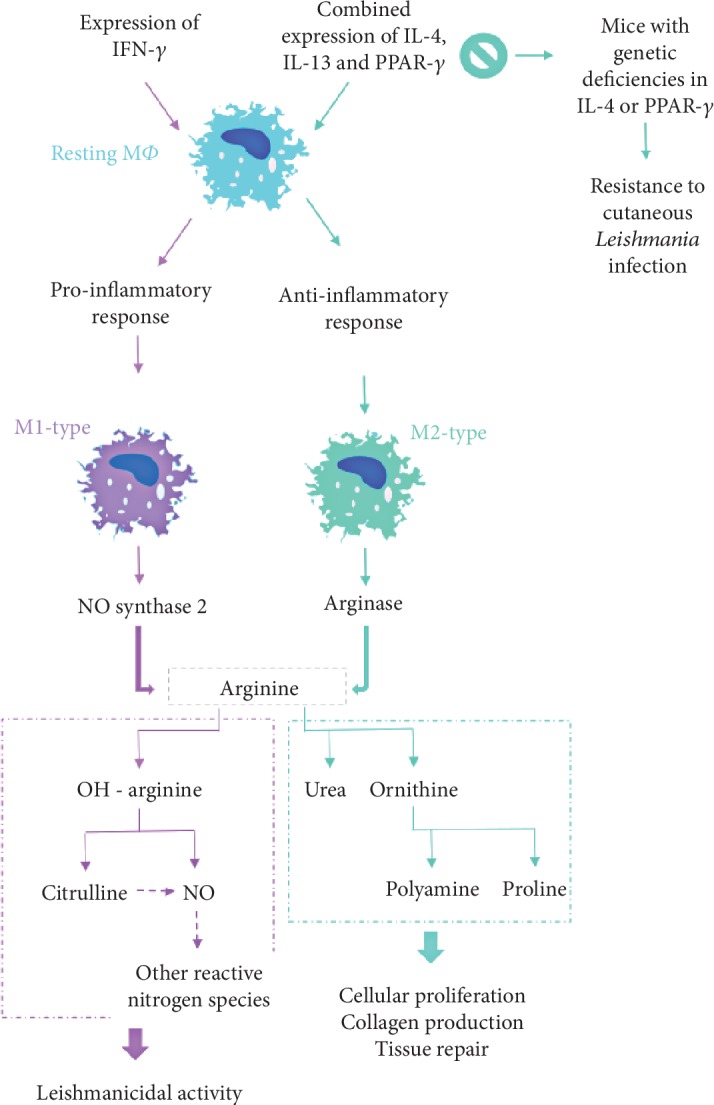
Differentiation of macrophage effector mechanisms against cutaneous species of *Leishmania* influences parasite fate and disease severity. IL: interleukin, IFN: interferon, M*Φ*: macrophages, NO: nitric oxide, PPAR: peroxisome proliferator-activated receptor.

Promastigotes are also taken up by M*Φ* that are the final host cells for *Leishmania* parasites [56]. In spite of being the parasite preferential host cells, since it is inside M*Φ* that replicates, these cells still are an immune barrier that parasites must overcome to persist in the host [26, 30]. Within M*Φ* phagolysosome, promastigote forms undergo morphological differentiation into small and nonmotile amastigotes able to resist to host cell killing mechanisms and survive under mammal high temperature (when compared to the sand fly) [30, 56]. Amastigotes replicate and promote the chronicity of cutaneous infection within dermal M*Φ* [30, 56]. *Leishmania* parasites can induce M*Φ* differentiation into two distinct phenotypes: M1 and M2 [30] (Figure 3). M1-M*Φ*, also called classically activated M*Φ*, are stimulated by the proinflammatory cytokines IFN-*γ* and TNF-*α*. These cytokines induce the expression of nitric oxide (NO) synthase 2 (NOS2), which degrades arginine into OH-arginine and subsequently into NO and citrulline [31, 66, 67]. NO can be further metabolized to other reactive nitrogen species, while citrulline can enter in the citrulline–NO cycle and synthesize NO [66–68]. This mechanism is responsible for NO-dependent leishmanicidal activity, which plays a key role against *Leishmania* infection [67, 68]. Although M1-M*Φ* response usually leads to parasite control, it also promotes necrosis of cutaneous lesions in consequence of an intense immune response that favors the development of severe wounds [69].

M2-M*Φ*, also known as alternatively activated M*Φ*, can be induced by different immunomodulators, including M*Φ* colony-stimulating factor (M-CSF), interleukin- (IL-) 4, and IL-1. According to stimulation, M2-M*Φ* can be phenotypically identified as M2a, M2b, M2c, or M2d, which seems to be involved in different immune activities.

M2-M*Φ* activate the arginine pathway by expressing arginase, an enzyme that hydrolyzes arginine into urea and ornithine. Ornithine is further metabolized into polyamine and proline, which induce cellular proliferation, collagen production, and tissue repair [68–70].

Moreover, in 2007, Odegaard et al. [71] reported that peroxisome proliferator-activated receptor- (PPAR-) *γ* has a role in the maturation of alternatively activated M*Φ* and showed that disruption of PPAR-*γ* in myeloid cells prejudices M2-M*Φ* activation [71, 72]. In fact, several studies obtained evidence that M2-M*Φ* are associated with *Leishmania* infection. In the case of CL, the involvement of PPAR-*γ* in M2-M*Φ* activation was only reported to *L. major*, *L. amazonensis*, and *L. mexicana* infection [30, 37, 69].

Although activation of M1-M*Φ* by both IFN-*γ* and TNF-*α* results in control of infection, this observation might not be a true predictor of disease progression to all cutaneous species of *Leishmania*, since it is also reported that IFN-*γ*-activated M*Φ* (M1-M*Φ*) are not able to incapacitate *L. amazonensis* amastigotes, favoring parasite survival [30, 50]. In resistant mice, *L. major* infected M*Φ*s will trigger T helper 1 (Th1) cells, directing the production of IFN-*γ* and, consequently, activating the inducible nitric oxide synthase (iNOS) and leading to differentiation of M1-M*Φ* [66, 73]. This will lead to parasite inactivation and slow down parasite dispersion [74].

Furthermore, in patients with diffuse CL due to *L. amazonensis*, arginase, polyamines, and prostaglandin E2, a lipid compound derived from fatty acids commonly associated with inflamed tissues seems to lessen local inflammatory immune response when compared with patients with local CL [75]. Thus, it is possible that together, these mediators favor the development of diffuse CL.

More recent studies have demonstrated that the arginase of *L. amazonensis* can mediate the posttranscriptional regulation of M*Φ* microRNAs [75]. The absence of parasitic arginase seems to favor NOS2 upregulation and the consequent increase of NO production, which can lead to parasite inactivation. Besides, a further study by Badirzadeh et al. did not found significant correlations between the activity of *L. major* arginase and the number, size, and duration of patient lesions [76, 77]. On the other hand, in the susceptible mice, IL-4, IL-6, and IL-10 were responsible for the suppression of iNOS-associated mechanisms and differentiation of M2-M*Φ* [30, 78].

Dendritic cells (DCs) and M*Φ* are important APCs that establish a bridge between innate and adaptive immunity. These cells present foreign antigens to helper T cells through class II molecules of major histocompatibility complex (MHCII) while these and other cell types can present antigens to cytotoxic T cells through class I molecules of major histocompatibility complex (MHCI). In addition to MHC molecules, costimulatory molecules are essential for T cell suitable activation. Downregulation of costimulatory molecules leads to impairment of signaling pathways and defective immune responses [79].

Several studies have shown that DCs can play a dichotomic functional role in the modulation of the host immune response, affecting the adaptive immune response and the disease outcome [78]. In *L. amazonensis* and *L. braziliensis* cutaneous lesions, dermal DCs (also called Langerhans cells) play distinct roles. In *L*. *amazonensis* patients, DCs are related to a Th2-type immune response, while in *L. braziliensis* patients, DCs are associated with a protective Th1 immune response [79].

On the other way, after *in vitro* restimulation, monocytes isolated from patients with CL caused by *L. braziliensis* showed a low expression of costimulatory molecules B7-1 (CD80) and B7-2 (CD86). Since costimulatory molecules are crucial for a proper T cell activation, *L. braziliensis* parasites appear to be able to regulate the patient immune response [80].

Like M*Φ*, *Leishmania* also interferes with intracellular signaling in DCs [73, 78]. Modulation of DC activation by *Leishmania* parasites appears to be species-specific [30, 78]. Experimental studies with *L. major*, *L. mexicana*, *L. amazonensis*, and *L. braziliensis* suggested that migratory DCs increase the expression of MHC and costimulatory molecules [30, 73, 78]. Histopathological observations of human skin biopsies reveal variation in severity of the skin lesions, which may be related to the density levels of DC subsets exhibiting diverse phenotype that by directing the T cell immune response may affect disease severity [3, 81, 82] (Table 2).

In experimental models, DCs process and provide the first contact of *Leishmania* antigens to T cells, leading to a preferential stimulation of IFN-*γ* produced by CD4^+^ T cells [30, 82, 83]. On the other hand, other studies performed in *L. major-*infected mice place in evidence that dermal DC may induce the expansion of the Treg cell subset [84].

Although the role of DCs in CL is very complex, these cells can be used to develop novel avenues that can lead to the generation of alternative therapies and therapeutic vaccines to improve the treatment of infected patients [85].

DCs and M*Φ*s express PRRs that allow parasite detection on host skin. TLRs are the most studied, and some of them, such as TLR3, TLR4, TLR7, and TRL9 have shown to play a key role in innate sensing and recognition of *Leishmania* by those cells [81]. This recognition is crucial to initiate the inflammatory response and control of parasite replication [30, 80].

After *in vitro* restimulation with parasite antigens, monocytes isolated from patients with LC caused by *L. braziliensis* showed upregulation of TLR9. Furthermore, it was found an association between the higher frequency of TLR9^+^ monocytes and lesion severity. The intracellular sensor TLR9, a transmembrane protein of endosomal compartments, binds to pathogen DNA triggering signal pathways that lead to a proinflammatory response. Thus, the upregulation of this sensor in CL patients points towards a release of proinflammatory cytokines, which do not seem to have a beneficial effect in reducing disease severity [80].

### 3.5. Interaction of Proinflammatory and Regulatory Lymphocyte Subsets Seems to Be the Hallmark of Cutaneous Leishmaniasis

B and T cells are key components of acquired immunity. B cells are responsible for generating antigen-specific antibodies (humoral response), and since they are APCs, they can also play a role in the activation of T cells [86–88]. These cells become activated after exposure to foreign antigens, which are internalized leading to the replication and differentiation of effector B cells and antibody released [30, 86]. Several studies suggest that B cells might be involved in the exacerbation of *Leishmania* infections, including cutaneous disease caused by *L. tropica*, *L. mexicana*, *L. major*, *L. braziliensis*, and *L. amazonensis*, though the mechanism behind it is still unknown [87, 89, 90].

However, studies performed with *L. major* in resistant rodent models (C3H/HeN and C57BL/6) suggest that B cells might have a role in the development of immunity against *Leishmania* infection [91, 92]. Furthermore, Mukbel et al. [93] showed that soluble factors, like species-specific immunoglobulins, and both CD4^+^ T and B cells derived from *L. major*-infected mice that healed the infection played a key role in killing *L. amazonensis* intracellular parasites. Also, a more recent study reported by Gibson-Corley [94] showed that B cells from C3HeB/FeJ mice coinfected with *L. major* and *L. amazonensis* promote parasite killing while B cells from coinfected C57BL/6 mice were ineffective in controlling infection. Although, action mechanism of B cells is not well elucidated, these findings may indicate that B cells can be a good target for development of a therapeutic for dermal leishmaniasis, since these cells seem to have a role in CL control in resistant mouse models [95].

**Figure 4:**
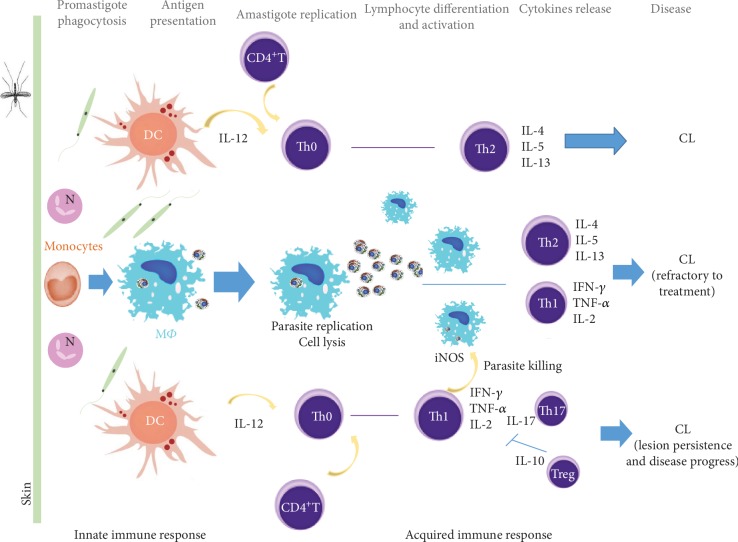
Activation of host immunity by cutaneous species of *Leishmania*. After skin infection, *Leishmania* promastigotes are uptake by phagocytes. IL-12 is secreted by activated DCs, and parasite antigens are presented by APCs, resulting in lymphocyte activation and secretion of proinflammatory (IL-2, IFN-*γ*, and TNF-*α*) cytokines that can activate M*Φ* microbicide mechanisms, leading to parasite inactivation. When IL-4, IL-5, and IL-13 predominate parasite replicates, allowing disease establishment. Differentiation of Th17 lymphocytes lead to a strong inflammatory environment that could cause tissue damage. Regulatory T cells inhibit lymphocyte activity promoting immune homeostasis and favoring disease progress. CL: cutaneous leishmaniasis; DC: dendritic cells, IFN: interferon; IL: interleukin; M*Φ*: macrophages; N: neutrophils; NO: nitric oxide; Th: T helper cell; Th0: naïve T cells; TNF: tumor necrosis factor; Treg: regulatory T cells.

The control of *Leishmania* infection and disease progression has long been associated with the generation of proinflammatory and anti-inflammatory immune response [30]. A sustained Th1 response characterized by elevated IL-12, IL-2, IFN-*γ*, and TNF-*α* and downmodulation of IL-4 and IL-10 production promotes M*Φ* activation (Figure 4) and seems to be crucial for host control of *Leishmania* parasite burden and clinical cure [30, 82, 83].

On the other hand, Th2-related cytokines (IL-4, IL-5, IL-10, and IL-13) inhibit M*Φ* activation, contributing to parasite survival [30, 83, 85].

In some CL clinical forms, a mixed Th1/Th2 immune response occurs during active infection, tending Th2 response to be dominant when both types of responses are activated [10]. The expansion of Th2 response is associated with the progression and chronicity of cutaneous lesion that is frequently refractory to classical leishmanial treatment, leading to severe mutilations [10, 83, 85].

Studies evaluating the production of cytokines in CL patients caused by *L. guyanensis* have shown high levels of IL-2 and IFN-*γ* [7]. In lesions, it has been detected a high density of Th2-related cytokines, particularly of IL-13. Moreover, these patients also can exhibit reduced or nondetectable antigen recognition associated with high levels of IL-10 and IL-5 and also lower specific antibody titers. In conclusion, enhanced Th2-type cytokines, which restrain Th1-type response, lead to an immune environment permissive to parasite replication [7, 96, 97]. Thus, *L. guyanensis* infection affects the expansion of antigen-specific T lymphocyte clones, causing low lymphocyte proliferation and decreasing IFN-*γ* production. Limited cellular and humoral responses during *L. guyanensis* infection may explain a high parasitic load and the recurrence of the disease [7].

Advances in the understanding of CL progression indicate that cellular interactions are more complex than the Th1/Th2 paradigm. Cutaneous *Leishmania* infection follows a complex set of interactions that can lead to the differentiation of the Th17 cell subset, which is characterized by releasing IL-17 [40, 98]. This cytokine, recognized as a proinflammatory modulator, induces other cells to release inflammatory mediators that ultimately promote PMN recruitment to the infection site, sustaining an inflammatory environment that can be associated with lesion persistence.

LC patients infected with *L. amazonensis* or *L. guyanensis* exhibited higher levels of Th17 lymphocytes [40]. In human infections caused by *L. major*, *L. tropica*, *L. amazonensis*, *L. braziliensis*, *L. guyanensis*, or *L. panamensis* was observed a high IL-17 production, pointing towards PMN recruitment and M*Φ* activation, which can be related to disease development and lesion severity [40, 99]. In murine studies, Th17 cells were associated with tissue destruction [40, 51, 88, 100].

Regulatory T (Treg) lymphocytes are considered a crucial cell subset to control the exacerbated inflammatory response, regulating disease pathology. The role of Tregs in the spectrum of CL immune responses varies across *Leishmania* species [40, 88, 100]. In *L. major*-infected mice, Tregs play varying roles in the disease outcome depending upon the genetic background and susceptibility of the host [101]. However, in the case of *L. panamensis* infection, Treg cells play a role in downregulating inflammatory cytokines, which appear to limit host cell recruitment leading to reduction of lesion size [101]. Hence, the role of Tregs and also of Th17 in CL is unclear, warranting further investigations able to evaluate cell dynamic and better understand the potential of being used as biomarkers of disease severity and a target for drug treatment.

*L. major* and *L. braziliensis in vivo* studies bring evidences that IL-10 subfamily is essential for the wound healing process, maintaining skin repair properties and limiting pathology independent of parasite control [85]. Other regulatory mechanisms mediated by cytokines must be explored in future studies for the control of *Leishmania*-induced immunopathology [102, 103].

Furthermore, it was reported that CL patients infected by *L. braziliensis* can present CD4^−^CD8^−^ (DN) T lymphocytes expressing *αβ* T cell receptors (TCR) and DN T cells expressing *γδ* TCR. While *αβ* DN T cell subset was associated with a more inflammatory environment, leading to antiparasitic activation of M*Φ*, *γδ* DN T cells seem to play a regulatory role, favoring the reduction of inflammatory response [103].

In CL, a proper local inflammatory immune response is crucial to contain and reduce parasite expansion. However, an extensive or excessive inflammatory response can cause tissue damage. Thus, the simultaneous finding of proinflammatory and regulatory cell subpopulations in CL patients seems to contribute to the balance between a protective immune response against the parasite and the natural intrinsic response that ensure the immune homeostasis.

NK T (NKT) cells, a specialized subpopulation of T lymphocytes, also seem to play a role during the early stages of *Leishmania* infections [91, 104, 105] (Figure 4). However, in *L. major*-infected mice, it was demonstrated that NKT cells appear to control parasite burden in skin lesions and in the spleen, but not in the lymph nodes [54], pointing towards an organ-specific role for these cells. Moreover, in the dermal lesions of *L. braziliensis-*infected patients, NKT cells with cytotoxic activity were identified [104].

## 4. Challenges of Prevention, Diagnosis, and Treatment of Cutaneous Leishmaniasis

Minimizing CL impact relies on prevention, diagnosis, and treatment, the three primary steps that must integrate developing new strategies for effective leishmaniasis control.

Prevention is the first pillar that needs to be ensured to achieve control of the disease. It encompasses (i) the reduction of population exposition to parasite vector, (ii) measures to diminish the role of vector and reservoirs in parasite transmission, (iii) awareness and education of the population at risk for this parasitic disease, (iv) availability of basic health care, and (v) monitoring of disease spread and incidence.

Vector control relies mostly on the use of insecticides, besides being toxic and needing a regular reapplication, which is a hassle for populations with low income and also generate a significant increase of insecticide-resistant vectors. Furthermore, the main reservoirs of the parasite are silvatic animals that can interact with the populations, which also increase the difficulty in controlling these parasites.

Monitoring of CL still is not an easy task since social stigma, war, poverty, and scarcity of access to health care largely affects most of the endemic regions that are associated with lack of equipment and of trained staff further aggravate these difficulties.

The diagnosis and treatment are interconnected, since the stage of disease progression and diagnosis accuracy highly influence the treatment efficacy. An early and accurate diagnosis and effective patient management are essential to reduce parasite transmission and CL increase.

In CL endemic areas, the accuracy of diagnosis must be made in the earlier clinical presentation to avoid the complications of advanced disease [106]. Specific approaches to treat CL patients have to take in consideration the etiologic agent, patient immune competence, clinical features, and arising complications in the course of *Leishmania* infection [11, 19]. Atypical infections may require an accurate differential diagnosis with other possible coexisting infections, such as leprosy, tuberculosis, fungal infections, ecthyma, furuncle, carbuncle, North American blastomycosis, paracocciomycosis, yaws, prototheca infection, condyloma acuminate, sporotrichosis, syphilis, lupus vulgaris, cutaneous furuncular myiasis, tungiasis, xanthoma tuberosum, sarcoidosis, pyoderma gangrenosum, and neoplasm [107]. Conventionally, the prompt CL diagnosis is obtained by the identification of amastigotes forms (round intracellular forms with 1.5 *μ*m to 3 *μ*m) of *Leishmania* in biopsy samples of skin lesion (gold standard) by optical microscopic observation [1]. Other methodologies may be either applied to the diagnosis of leishmaniasis, like skin histological analyses, *in vitro* biopsy culture, and molecular diagnosis [25]. Occasionally, the leishmanin skin test (LST), also called the Montenegro skin test (MST) and delayed-type hypersensitivity (DTH), is used in CL as a marker of cellular immune response (Table 2). When CL diagnosis has been unequivocally established, it is necessary to apply specific targeted therapy and manage the patient to control the infection [12, 14]. In some cases, it is necessary to monitor adverse effects including myalgia, gastrointestinal disturbances, headache, anorexia, asthenia, fever, neurological alterations, and arrhythmia and also to use the medical imaging techniques, like magnetic resonance imaging (MRI) to show no cartilaginous destruction or paranasal involvement in severe cases [12, 108].

Antileishmanial drugs applicable to CL are limited and display severe side effects, elevated costs, and usually require prolonged treatments [109]. The species of *Leishmania* involved in the infection, parasite resistance, and concomitant infections are key factors that influence the efficacy of the treatment [110, 111]. Other treatment possibility is the use of thermotherapy; nevertheless, this technique is not widely available due to the cost of the devices and procedures required and the need of skilled health professionals to perform the treatment [112]. Recent studies about the specific interventions to treat CL in children provide evidences of the scarcity of data available to support treatment recommendations for this age range and of the unmet need to develop and test better treatment options for this vulnerable group [113].

The availability of effective, low cost, and safe treatments like prophylactic vaccines, drugs and therapeutic vaccines for cutaneous infection is not yet available for human leishmaniasis. Furthermore, other insurgent issues such as climate changes, migration of populations, and permissiveness of vectors can make more complex CL control, promoting disease spreading [16]. Climate changes can lead to the spreading of the vectors to nonendemic regions [1, 114]. The dispersion of the vector allied with high vectorial capacity and permissiveness can facilitate the adaptation of the parasite in nonendemic areas, leading to the generation of new foci and increasing the risk of parasite transmission [3, 115, 116]. Furthermore, economic problems, natural disasters, and wars associated with mass migration and tourist travellers can lead to an increased risk of infection exposure [4, 20, 117].

## 5. Closing Remarks

Cutaneous leishmaniasis is an important public health problem worldwide. The spectrum of *Leishmania* infection can be subclinical, localized and disseminated, and relies on the immune competence of the host and on the infectivity of parasite species.

The fact of CL present high incidence mainly in areas with lack of economic resources, insufficient trained health professionals, and low awareness for the health issue of leishmaniasis allied with the lack of highly effective vector and reservoir control, treatments and no availability of a vaccine, creates an environment that promotes the CL propagation, turning the disease in pressing concern global health.

Together with the human description of the specific immune response, animal models have been used to extensively characterize the immune response to parasite infections caused by cutaneous species of *Leishmania.* Therefore, the investment in CL additional studies is urgent and essential, underlying the factors regulating immune pathological responses, which are needed for the implementation of more efficient and integrated control strategies and therapeutics. These efforts are indispensable for the populations affected by the disease, which are in desperate need of affordable and effective alternatives to the available treatments that are associated with parasite resistance and severe toxic effects.

## Figures and Tables

**Table 1:** Old and New World species of *Leishmania* causing cutaneous leishmaniasis and their respective geographic distribution, vectors, hosts, and reservoir, adapted from [2, 5–7, 15].

Species	Geographic distribution	Vectors	Transmission cycle	Hosts	Reservoirs
*L. (L.) aethiopica*	Ethiopia	*Ph. longipes*, *Ph. pedifer*	Zoonotic	Rodents, wild canids, dogs	Hyraxes
*L. (L.) major*	Asia & Africa	*Phlebotomus papatasi*, *Ph. duboscqi*, *Ph. salehi*	Zoonotic	Small rodents, dogs, humans	Small mammals and birds
*L. (L.) tropica*	Asia, Africa and Mediterranean	*Ph. sergenti*	Predominant anthroponotic	Rodents, wild canids, dogs	Humans, hyraxes
*L. (L.) amazonensis*	South America	*Lu. flaviscutellata*, *Lu. reducta*, *Lu. olmeca olmeca*, *Lu. nuneztovari*	Zoonotic	Terrestrial forest rodents, marsupials, wild canids, humans	Rodents, edentates, marsupials, wild canids
*L. (L.) garnhami*	Venezuelan Andes	*Lu. youngi*	Zoonotic	The opossum *Didelphis marsupialis* and humans	Marsupials
*L. (L.) mexicana*	USA (Texas), Central and South America	*Lutzomyia olmeca olmeca*, *Lu. diabolica*, *Lu. anthophora*, *Lu. columbiana*, *Lu. ayacuchenisis*, *Lu. ylephiletor*, *Lu. cruciata*, *Lu. longipalpis*	Zoonotic	Rodents: *Ototylomys phyllotis*, *Nyctomys sumichrasti*, *Heteromys desmarestianus*, *Sigmodon hispidus*, *Neotoma albigula*, *Proechimys* sp.*, Oryzomys* sp., *Nectomy* sp.*, Neacomys* sp., *Dasyprocta* sp.; *marsupials*: *Marmosa* sp.*, Metachirus* sp.*, Didelphis* sp.*, Philander* sp.; wild canids: *Cerdocyon thous*; humans	Rodents, edentates, marsupials
*L. (L.) pifanoi*	Apparently limited to Venezuela	*Lu. flaviscutellata*, *Lu. olmeca bicolor*	Zoonotic	Humans. Wild rodents, *Rattus rattus*	Rodents
*L. (L.) venezuelensis*	Venezuela, in the states of Lara and Yaracuy	*Lu. olmeca bicolor*, *Lu. rangeliana*	Zoonotic	Cats and humans	Rodents: *Sigmodon hispidus*, *Rattus rattus*
*L. (L.) shawi*	Brazilian Amazon region	*Lu. whitmani complex*	Zoonotic	Monkeys: *Cebus apella*, *Chiropotes satanas*; edentates: *Choloepus didactylus*, *Bradypus tridactylus*; procyonids: *Nasua nasua*; humans	Monkeys, edentates, procyonids
*L. (V.) braziliensis*	Central & South America	*Lu. intermedia*, *Lu. whitmani*, *Lu. wellcomei*, *Lu. migonei*, *Lu. neivae*, *Lu. davisi*, *Lu. ovallesi*, *Lu. carrerai carrerai*, *Lu. spinicrassa*, *Lu. trapidoi*, *Lu. gomezi*, *Lu. ylephiletor*, *Lu. umbralitis, flaviscutellata*, *Lu. olmeca*	Zoonotic	Rodents: *Oryzomys concolor*, *O. capito*, *O. nigripes*, *Akodon arviculoides*, *Proechimys* sp., *Sigmodon hispidus*, *Bolomys lasiurus*, *Rhipidomys leucodactylus*, *Rattus rattus*; marsupials: *Didelphis marsupialis*; dogs, cats, and horses	Humans. Terrestrial rodents and some marsupials
*L. (V.) colombiensis*	Colombia, Panama, Venezuela, forests of Brazil and Peruvian lowlands, others Latin American countries	*Lu. hartmanni*, *Lu. gomezi*, *Lu. panamensis*	Zoonotic	Sloth *Choloepus hoffmanni* and humans	Edentates
*L. (V.) guyanensis*	South America	*Lu. umbratilis*, *Lu. anduzei*, *Lu. Ovallesi*, *Lu. whitmani*	Zoonotic	Rodents, edentates: *Choloepus didactylus*, *Tamandua tetradactyla*; marsupials, humans	Rodents, edentates, marsupials
*L. (V.) lainsoni*	Forested areas of Brazil, Peru, and Bolivia	*Lu. ubiquitalis, Lu. velascoi*	Zoonotic	Rodents *Agouti paca* and humans	Rodents
*L. (V.) lindenbergi*	Degraded forest in Belém, Pará, Brazil	*Currently unknown Lu. antunesi is highly suspected*	Zoonotic	Humans	It is suspected that the wild animal reservoirs are probably terrestrial
*L. (V.) naiffi*	States of Pará and Amazonas (Brazil), French Guyana	*Lu. ayrozai*, *Lu. paraensis*, *Lu. squamiventris*	Zoonotic	Nine-banded armadillo *Dasypus novemcinctus*, humans	Edentates
*L. (V.) panamensis*	Central America	*Lu. trapidoi*, *Lu. ylephiletor*, *Lu. gomezi, Lu. panamensis*, *Lu. hartmanni*	Zoonotic	Rodents: *Heteromys* sp.; edentates: *Choloepus hoffmanni*, *Bradypus infuscatus*, *B. griseus*; marsupials, procyonids: *Bassaricyon gabbi*, *Nasua nasua*, *Potos flavus*, *monkeys*: *Aotus trivirgatus*, *Saguinus geoffroyi*; hunting dogs, humans	Rodents, edentates, marsupials, procyonids, monkeys
*L. (V.) peruviana*	South America	*Lu. peruensis, Lu. verrucarum*	Zoonotic	Humans and dogs. Rodents: *Phyllotis andinum*; marsupials: *Didelphis marsupialis*	Rodents, marsupials

**Table 2:** Clinical presentation and delayed-type hypersensibility (DTH) of cutaneous *Leishmania* species in the world, adapted from [2, 82, 90].

Subgenus	Species	Main clinical presentation	DTH (skin test)
*Leishmania*	*L. major*	Localized	—
	*L. tropica*	Localized	DTH +
	*L. aethiopica*	Localized	—
	*L. mexicana*	Localized Borderline disseminated Anergic diffuse	DTH + DTH – DTH –
	*L. amazonensis*	Localized Borderline disseminated Anergic diffuse	DTH – DTH – DTH –

*Viannia*	*L. panamensis*	Localized LC Borderline disseminated Mucocutaneous	DTH + DTH – DTH ++++
	*L. braziliensis*	Localized Borderline disseminated Mucocutaneous	DTH + DTH – DTH ++++
	*L. peruviana*	One or few lesions	—
	*L. venezuelensis*	Single and multiple skin lesions Disseminated nodules (confused with diffuse)	— —
	*L. pifanoi*	Diffuse	—
	*L. guyanensis*	Single and multiple skin lesions Rare cases of mucocutaneous	— —
	*L. shawi*	Single and multiple skin lesions Cases of multiple lesions, clearly due to metastases, are occasionally seen	— —
	*L. colombiensis*	Single and multiple skin lesions	—
	*L. naiffi*	Localized	—
	*L. lainsoni*	Localized	—
	*L. lindenbergi*	Localized	—
	*L. garnhami*	Localized	—
